# Hazards of Healthy Living: Bottled Water and Salad Vegetables as Risk Factors for Campylobacter Infection

**DOI:** 10.3201/eid0910.020823

**Published:** 2003-10

**Authors:** Meirion R. Evans, C. Donald Ribeiro, Roland L. Salmon

**Affiliations:** * University of Wales College of Medicine, Cardiff, United Kingdom; † Cardiff Public Health Laboratory, Cardiff, United Kingdom; ‡ Public Health Laboratory Service Communicable Disease Surveillance Centre (Wales), Cardiff, United Kingdom

## Abstract

Campylobacter is the most common cause of bacterial gastroenteritis worldwide, yet the etiology of this infection remains only partly explained. In a retrospective cohort study, we compared 213 sporadic campylobacter case-patients with 1,144 patients with negative fecal samples. Information was obtained on food history, animal contact, foreign travel, leisure activities, medical conditions, and medication use. Eating chicken, eating food from a fried chicken outlet, eating salad vegetables, drinking bottled water, and direct contact with cows or calves were all independently associated with infection. The population-attributable fractions for these risk factors explained nearly 70% of sporadic campylobacter infections. Eating chicken is a well-established risk factor, but consuming salad and bottled water are not. The association with salad may be explained by cross-contamination of food within the home, but the possibility that natural mineral water is a risk factor for campylobacter infection could have wide public health implications.

Campylobacter is the most commonly reported bacterial cause of foodborne infection in the Western world and affects more than 2 million people in the United States each year (*1*). In England and Wales, over 50,000 campylobacter cases are reported each year and show no signs of a decline in incidence (*2*). For every case reported to laboratory surveillance, another seven cases are estimated to occur in the community, suggesting that from 0.5% to 1.0% of the United Kingdom’s population is infected annually (*3*). Although the infection usually causes a mild, self-limiting illness, serious sequelae, including Guillain-Barré syndrome and death, occur in approximately 1 in 1,000 and 1 in 20,000 infections, respectively (*1*). Many national food safety agencies, such as the Food Standards Agency in the United Kingdom, have set goals of reducing food poisoning. To achieve these goals, a much clearer understanding of the etiology of campylobacter infection will be necessary.

In spite of the frequency of campylobacter infections, the cause has proved elusive. Recognized outbreaks are rare and are usually caused by contaminated water, milk, or poultry (*4*,*5*). However, these food products explain only a small proportion of sporadic cases, and the source of infection is unaccounted for in >60% of U.K. campylobacter cases (*6*,*7*). Several case-control studies of risk factors for sporadic campylobacter infection have been performed in the United Kingdom (*6*–*10*), but many unanswered questions remain. We conducted a retrospective cohort study that involved mailing a questionnaire to the patient at the time the fecal specimen was received by the laboratory to investigate the cause of sporadic campylobacter infection in the community.

## Methods

The study population included all persons living in the Cardiff area who consulted their general practitioner for gastrointestinal symptoms and subsequently submitted a diagnostic fecal sample for microbiologic testing from January 1 through December 31, 2001. Cardiff Public Health Laboratory is the sole laboratory providing a diagnostic microbiology service for the area. All specimens were cultured for *Campylobacter* spp*.*, *Salmonella* spp, *Shigella* spp., and *Escherichia coli* O157 and examined for ova and parasites, by standard methods. Follow-up specimens from the same patient (<4 weeks after the previous specimen submission date); specimens received from hospital wards and other sites were excluded from study. The study was approved by the local research ethics committee.

Immediately upon receipt of the specimen at the laboratory (next working day), a questionnaire, together with an explanatory letter and a postage-paid envelope, was mailed to the patient. Patients who had not responded within 1 week were sent a reminder letter and provided with another questionnaire on request. The questionnaire asked about basic personal details, including age, sex, employment status, occupation, details of illness, and details of household contacts. It included sections on foreign travel, food and drink eaten, animal contact (pets and farm animals), outdoor leisure activities (gardening, walking, visits to parks or farms, fishing, swimming, and sports), and questions on specific medical conditions and medication (antacids, H2 antagonists, and antibiotics). The food history covered meat and fish, poultry and eggs, vegetables (raw vegetables, leaf vegetables [e.g., lettuce], salad vegetables [e.g., tomato], and prepared salads [e.g., coleslaw]), fruit, milk and dairy products, drinking water (tap water, bottled water, and other sources), and eating out (type of restaurant or takeaway). Participants were asked to respond yes or no and, to the question of exposure for tap water, to indicate the number of glasses drunk per day. All questions related to exposure in the 7 days before the onset of symptoms, except for those on antibiotics, which concerned the month before illness onset.

Case-patients were defined as any patient, not associated with an outbreak, who submitted a fecal sample that was positive for *Campylobacter* spp. on microbiologic culture. Case-patients were compared with patients whose samples were negative on culture and microscopic examination. Patients with an alternative microbiologic diagnosis were excluded (unless they had dual infection with campylobacter).

Initial univariate analysis was performed with Epi Info software (v. 6.04; Centers for Disease Control and Prevention) to calculate maximum likelihood estimates for Mantel-Haenzel odds ratios (OR) with exact 95% mid-p confidence limits. Continuous variables were analyzed using the t test or Mann-Whitney U test, as appropriate. All reported p values are two sided. Multiple logistic regression models were constructed with Stata software (v. 6, Stata Corporation, College Station**,** TX). Risk factors were selected a priori on biologic grounds and grouped into four exposure categories: food and drink consumption; animal contact; leisure activities, including foreign travel; and medical history. Logistic regression models were first constructed for risk factors within each exposure category (adjustment A). We then fitted a model that combined all the independent risk factors (for which the Wald test p value for the adjusted OR was <0.10) from the four exposure categories (adjustment B). Finally, to detect any residual confounding, we fitted all personal factors with a p value of <0.10 (age group, presence of a child <5 years of age in the household, and employment status). Of these, only age group interacted significantly with the other terms as tested by goodness of fit and was therefore included in the final model (adjustment C). The population-attributable fraction for each risk factor associated with campylobacter infection was calculated by using methods described by Greenland and Drescher (*11*).

## Results

Questionnaires were sent to 2,694 eligible patients; fecal samples from 346 (12.8%) were positive for *Campylobacter* spp. (including 4 dual infections: 3 with salmonella infection, 1 with giardiasis). No campylobacter outbreak occurred during the study period. Ninety-one patients (3.4%) were positive for other organisms (42 *Salmonella* spp., 20 *Giardia lamblia*, 12 *Cryptosporidium* sp., 7 *Clostridium difficile*, 2 *Shigella* sp., 2 *E. coli* O157, 1 amoebic dysentery, and 5 other parasites); these were excluded from further analysis. Responses were received from 213 (61.6%) of 346 persons with campylobacter infection and 1,144 (50.7%) of 2,257 persons with negative specimen results. Median delay in response (from date questionnaire sent to date questionnaire returned) was 6 days (range 2–73 days) for case-patients and 7 days (range 1–77 days) for non-case-patients (Kruskal-Wallis H 1.81, p=0.18).

### Personal Factors and Symptoms

Case-patients (median 43 years of age, range 0–80 years of age) were significantly older than non-case-patients (median 36 years of age, range 0–100 years of age) (Kruskal-Wallis H 5.31, p=0.02) (Table 1). Non-case-patients were also more likely to come from a household that included a child <5 years of age (even after adjusting for the age of the respondent), although not more likely to report prior diarrheal illness in a household contact. Case-patients were more likely to report symptoms than non-case-patients, particularly fever (OR 3.19; 95% confidence interval [CI] 2.36 to 4.31), muscle aches (OR 3.13; 95% CI 2.32 to 4.22), and abdominal pain (OR 3.40; 95% CI 2.32 to 5.12. Nearly all case-patients and most non-case-patients had diarrhea, but case-patients (18.3%) were more likely than non-case-patients (11.8%) to report blood in the stool (OR 1.67; 95% CI 1.12 to 2.46).

**Table 1:** Comparison of personal and household factors in campylobacter case-patients and non-case-patients^a^

Variable	Case-patients (n=213)		Non-case-patients (n=1144)		OR (95% CI)	p value
	No.	(%)	No.	(%)		
Female	99	(46.5)	504	(44.1)	1.10 (0.82 to 1.48)	0.56
Age group						
0–14 y	26	(12.2)	328	(28.7)	Reference	
15–44 y	84	(39.4)	323	(28.2)	3.28 (2.06 to 5.23)	<0.001
45–64 y	72	(33.8)	231	(20.2)	3.93 (2.44 to 6.35)	<0.001
>65 y	30	(14.1)	255	(22.3)	1.48 (0.86 to 2.57)	0.16
Employment status						
Employed	92	(43.2)	352	(30.8)	Reference	
Full-time student	27	(12.7)	82	(7.2)	1.26 (0.77 to 2.06)	0.36
Caring for home and family	15	(7.0)	84	(7.3)	0.68 (0.38 to 1.23)	0.21
Other	45	(21.1)	453	(39.6)	0.38 (0.26 to 0.56)	<0.001
Unemployed	10	(4.7)	31	(2.7)	1.23 (0.58 to 2.61)	0.58
Long-term illness	16	(7.5)	84	(7.3)	0.53 (0.24 to 1.14)	0.29
Mean no. of other people in household (median, range)	3.0	(3) (1-12)	3.2	(3) (1-36)		0.98^b^
Child <5 y of age in the household	28	(13.1)	326	(28.5)	0.38 (0.25 to 0.57)	<0.0001
Mean no. of children <5 y of age in household (median, range)	0.21	(0) (0-5)	0.41	(0) (0-5)		<0.0001^b^
Other ill person in the household	15	(7.0)	112	(9.8)	0.70 (0.39 to 1.20)	0.26
Mean no. of other ill people in household (median, range)	0.09	(0) (0-3)	0.15	(0) (0-6)		0.20^b^
Child caregiver	3	(1.4)	25	(2.2)	0.64 (0.15 to 1.94)	0.61^c^
Food handler	8	(3.8)	61	(5.3)	0.69(0.31 to 1.41)	0.43

^a^OR, odds ratio; CI, confidence interval. ^b^Mann-Whitney U test. ^c^Fisher exact test.

### Food and Drink Consumed

Case-patients were more likely to report eating meat, including beef, pork, and ham; poultry products, including chicken and eggs; and a variety of uncooked vegetables and fruit, including lettuce, other salad vegetables (cucumber, tomatoes, etc.), preprepared salad (coleslaw, etc.), and fresh or frozen berries. An association existed with drinking bottled water (OR 1.98; 95% CI 1.48 to 2.67) and between infection and drinking cold tap water (OR 1.51; 95% CI 1.06 to 2.18) but not with drinking cold milk. Neither tap water nor milk consumption showed a dose response relationship. Case-patients were more likely to have eaten out in the 7 days before illness onset, particularly at a fried chicken outlet, sandwich bar, or other unspecified restaurant.

### Animal Contact, Leisure Activities, and Medical History

Case-patients were no more likely than non-case-patients to report pet ownership or contact with other people’s pets. Non-case-patients were more likely to own a pet rabbit, though this association was weaker after adjusting for age. Case-patients were more likely to have gone walking, to have visited a farm, or to report contact with cows or calves in the 7 days before illness, though the number of persons exposed to cows was very small. No difference existed in history of recent foreign travel. In respect to medical history, case-patients were no more likely than non-case-patients to suffer from diabetes or indigestion, or to be taking antacid or ulcer medicines but were less likely to report preexisting bowel disease or to have taken antibiotics in the month before onset of illness.

### Multivariate Analysis

After adjustment for other variables within each of the four exposure groups (adjustment A), several independent risk factors were identified (Table 2). After combining all these variables (adjustment B), eating frozen fish, eggs, or berries; having milk delivered to the home; eating out at a Chinese restaurant or takeaway; and walking were dropped from the model as they made no independent contribution to the outcome. In the final model (adjustment C), five variables were identified as independent risk factors for campylobacter infection: eating chicken, eating salad vegetables other than lettuce (e.g., tomatoes, cucumber), drinking bottled water, eating out at a fried chicken outlet, and contact with cows or calves (Table 3). Eating lamb, owning a pet rabbit, a history of lower bowel problems, and having had antibiotics in the month before illness all showed a protective effect. The combined population-attributable fraction for the five independent risk variables associated with campylobacter infection was nearly 70%. The highest attributable fractions were for eating chicken (31%), eating salad (21%), or drinking bottled water (12%).

**Table 2:** Frequency of food exposure, animal contact, leisure activities and medical history in campylobacter infected case-patients and non-case-patients

Exposure	Case-patients (%) (n=213)	Non-case-patients (%) (n=1144)	Crude OR^a^ (95% CI)	Adjusted OR (95% CI)	p value
Food and drink					
Lamb	47 (22.1)	282 (24.7)	0.87 (0.61 to 1.22)	0.67(0.45 to 0.99)	0.046
Frozen fish	53 (24.9)	341 (29.8)	0.78 (0.55 to 1.09)	0.64 (0.45 to 0.93)	0.020
Chicken	177 (83.1)	777 (67.9)	2.32 (1.60 to 3.43)	1.61 (1.03 to 2.50)	0.036
Eggs	141 (66.2)	606 (53.0)	1.74 (1.28 to 2.37)	1.35 (0.95 to 1.92)	0.096
Salad vegetables	159 (74.6)	635 (55.5)	2.36 (1.70 to 3.30)	1.73 (1.09 to 2.74)	0.019
Fresh or frozen berries	51 (23.9)	173 (15.1)	1.77 (1.23 to 2.51)	1.43 (0.95 to 2.13)	0.086
Milk delivered to the doorstep	29 (13.6)	215 (18.8)	0.68 (0.44 to 1.03)	0.60 (0.38 to 0.94)	0.026
Bottled water	114 (53.5)	420 (36.7)	1.98 (1.48 to 2.67)	1.39 (0.98 to 1.96)	0.062
Ate at a fried chicken outlet	22 (10.3)	51 (4.5)	2.47 (1.44 to 4.13)	1.82 (1.00 to 3.30)	0.050
Ate at a Chinese restaurant	23 (10.8)	114 (10.0)	1.09 (0.67 to 1.74)	0.58 (0.33 to 0.99)	0.048
Animal contact					
Own a pet rabbit	7 (3.3)	89 (7.8)	0.40 (0.17 to 84)	0.46 (0.20 to 1.05)	0.066
Had contact with cows or calves	5 (2.3)	6 (0.5)	4.55 (1.27 to 15.74)	5.44 (1.05 to 28.1)	0.043
Leisure activities					
Walking (>15 min)	162 (76.0)	712 (62.2)	1.93 (1.38 to 2.72)	1.92 (1.34 to 2.73)	<0.001
Medical history					
History of lower bowel problems	21 (9.9)	197 (17.2)	0.53 (0.32 to 0.83)	0.55 (0.34 to 0.90)	0.018
Antibiotic in month before illness	11 (5.2)	160 (14.0)	0.34 (0.17 to 0.61)	0.34 (0.18 to 0.65)	0.001

^a^Adjusted for potential confounders within each exposure group; OR, odds ratio; CI, confidence interval.

**Table 3:** Multiple logistic regression analysis showing independently associated variables in campylobacter case-patients and non-case-patients^a^

Variable	Adjustment A^b^		Adjustment B^c^		Adjustment C^d^		Attributable fraction (%) (95% CI)	
	OR (95% CI)	p value	Odds ratio (95% CI)	p value	OR (95% CI)	p value		
Lamb	0.67 (0.46 to 0.99)	0.05	0.69 (0.48 to 1.00)	0.05	0.68 (0.47 to 0.98)	0.04		
Chicken	1.61 (1.03 to 2.50)	0.04	2.01 (1.35 to 3.00)	0.001	1.79 (1.19 to 2.69)	0.005	31 (9 to 48)	
Salad vegetables	1.73 (1.09 to 2.73)	0.02	1.99 (1.40 to 2.82)	<0.001	1.53 (1.06 to 2.21)	0.02	21 (2 to 36)	
Bottled water	1.39 (0.98 to 1.96)	0.06	1.57 (1.15 to 2.14)	0.005	1.41 (1.02 to 1.95)	0.04	12 (0 to 23)	
Ate at a fried chicken outlet	1.82 (1.00 to 3.30)	0.05	2.08 (1.20 to 3.62)	0.01	1.94 (1.10 to 3.42)	0.02	4 (0 to 7)	
Had contact with cows or calves	5.44 (1.05 to 28.10)	0.04	3.98 (1.08 to 14.65)	0.04	5.07 (1.30 to 19.74)	0.02	1 (0 to 3)	
Own a pet rabbit	0.46 (0.20 to 1.05)	0.07	0.36 (0.16 to 0.81)	0.01	0.37 (0.16 to 0.83)	0.015		
History of lower bowel problems	0.55 (0.34 to 0.90)	0.02	0.48 (0.29 to 0.79)	0.004	0.45 (0.27 to 0.73)	0.001		
Had antibiotic in month before illness	0.34 (0.18 to 0.65)	0.001	0.41 (0.21 to 0.78)	0.006	0.40 (0.21 to 0.77)	0.006		

^a^OR; odds ratio; CI, confidence interval. ^b^Adjustment A, adjusted for potential confounders within each exposure group. ^c^Adjustment B, adjusted for other significant variables from all four exposure groups. ^d^Adjustment C, adjusted for age group.

## Discussion

Our study identified five risk factors for campylobacter infection that, if taken together, could account for most sporadic cases. Most important was eating chicken in the 7 days before onset of illness. Two other risk factors, not previously described, could also potentially account for a sizeable proportion of case-patients: eating salad vegetables such as tomatoes or cucumber and drinking bottled water.

The study used a retrospective cohort design that included all patients submitting fecal specimens through their general practitioner to a single laboratory. This design controls for patient characteristics associated with physician-consulting behavior and may also minimize recall bias associated with using healthy controls. Use of a laboratory study population does, however, have several disadvantages. Non-case-patients probably represent a group whose illnesses have disparate cause. Many may have had viral gastroenteritis since this is known to be common in the community and is not detectable by routine laboratory testing. This fact would explain why symptoms reported by non-case-patients were milder. Non-case-patients were also significantly more likely than case-patients to report a history of lower bowel problems, suggesting that some had pre-existing disease that might predispose to non-infectious diarrhea. Antibiotic use in the month before onset of illness was also more common in non-case-patients, and symptoms in these persons may therefore be a side effect of antibiotic treatment. Persons with pre-existing bowel problems may have atypical dietary habits, but neither a history of bowel problems nor of antibiotic use should affect the accuracy of food histories. The multivariate analysis controlled for both these variables.

The most consistent finding in studies of campylobacter infection etiology has been an association with eating chicken. This finding has been described in three previous U.K. studies (*6*,*7*,*9*), and in studies from the United States (*12*–*16*), Scandinavia (*17*–*19*), the Netherlands (*20*), Switzerland (*21*), and New Zealand (*22*,*23*). However, the relationship with chicken is sometimes only seen for eating undercooked chicken (*12*,*22*,*23*) or eating chicken away from home (*8*,*15*,*22*,*23*). Recent microbiologic studies of raw poultry in the United Kingdom indicate continuing high levels of campylobacter contamination in chicken and the occurrence of identical subtypes in both chicken and human isolates (*24*). Our finding of an association between illness and eating chicken or eating from a fried chicken outlet highlights the fact that chicken remains a major risk factor for campylobacter in the United Kingdom and that measures are needed both in the food industry and at home to promote thorough cooking of chicken and to reduce the risk for cross-contamination.

Neither eating salad vegetables nor drinking bottled water has previously been recognized as a risk factor. In our study, both these associations made a significant contribution to the final logistic regression model and could explain a substantial number of campylobacter infections. Both are also biologically plausible. Salad vegetables could be contaminated with campylobacter either before or after the point of sale. Contamination at the source could occur through contaminated soil or contaminated water during harvesting. Salad vegetables are often imported from abroad, and changes in the sourcing of such items could introduce new vehicles of campylobacter infection into the U.K. food chain. For example, contaminated imported lettuce has been identified as a vehicle of infection in recent salmonella and shigella outbreaks in the United Kingdom (*25*). However, recent extensive sampling of organic fruit and vegetables and ready-to-eat prepackaged salads in the United Kingdom found no pathogens such as *Campylobacter*, *Salmonella,* or *E. coli* O157, suggesting that contamination of such items is either rare or intermittent (*26*). Two reports on campylobacter outbreaks associated with salad have been published. Both of these involved contamination in the kitchen. The first was a 3-month long outbreak from cucumber served at a salad bar; the outbreak resolved after changes were made in food preparation and storage procedures (*27*). The second involved salad prepared by a foodhandler who exhibited symptoms of campylobacter infection (*28*). In a recent review of outbreaks in England and Wales (including five from campylobacter) linked with salad vegetables and fruit, cross-contamination was also the most frequently identified contributory factor (*25*). The association we observed was specific to items such as tomatoes and cucumber that require extensive handling during preparation and often the use of a chopping board, rather than with lettuce or with salads bought preprepared. This finding suggests that salad most likely gets cross-contaminated during food preparation.

Natural mineral water is usually obtained from springs and occasionally from borehole sources. In Europe, legislation requires that mineral water be free from parasites and pathogenic organisms but, unlike tap water, it may not be treated in any way that might alter its chemical composition (*29*). A variety of organisms, including coliforms, can be found in mineral water and will survive for a considerable length of time, particularly in uncarbonated water supplied in plastic bottles or bottled by hand (*30*). To our knowledge, campylobacter has not been identified in mineral water, but this may simply be because testing for campylobacter is rarely undertaken. Mineral water has, however, been identified in the past as a vehicle of transmission during a cholera epidemic (*31*) and as a potential source of typhoid fever in travelers (*32*). More recently, a study of diarrhea in HIV-infected persons found symptoms were significantly associated with drinking bottled or filtered water, whereas drinking tap water was protective (*33*). Drinking bottled water has also recently been identified as a possible risk factor for campylobacter infection acquired abroad (*34*), and for *Campylobacter coli* infection (*35*). These findings suggest that bottled water could, given the right circumstances, provide a vehicle of transmission for campylobacter.

A small proportion of cases were explained by contact of personswith cows or calves. This occurred exclusively within the context of a farm visit and probably reflects the urban context of our study. This association, though apparently uncommon, is entirely plausible. Occupational contact with animal feces (*8*), living on a farm (*16*,*19*), and contact with cattle (*16*,*23*) have all been previously described as risk factors for campylobacter infection. Healthy beef and dairy cattle both excrete campylobacter (*36*,*37*), and molecular evidence suggests a link between campylobacter in the farm environment with those causing disease in the community (*38*).

Our study confirms that eating chicken still plays an important role in the cause of campylobacter infection. It also identifies two potentially important new risk factors that merit further investigation: salad vegetables (and the associated risks of cross-contamination in the home) and bottled natural mineral water. Cross-contamination in the domestic kitchen is potentially preventable, but we need to know how it happens and what interventions are most effective at reducing the risk. Bottled water is a $35 billion worldwide industry (*39*). In the United States, 1.7 billion gallons of natural mineral water were consumed in 2000 (*39*). Consumption is also increasing dramatically in the United Kingdom (by approximately 10% each year), and approximately 300 million gallons of bottled water are now consumed annually (*40*). Consequently, increased illness from contamination of bottled water could be considerable. More studies of the microbiologic quality of natural mineral waters are required, and these should include testing for *Campylobacter* spp.
